# Crohn’s Disease Localization Displays Different Predisposing Genetic Variants

**DOI:** 10.1371/journal.pone.0168821

**Published:** 2017-01-04

**Authors:** Orazio Palmieri, Fabrizio Bossa, Maria Rosa Valvano, Giuseppe Corritore, Tiziana Latiano, Giuseppina Martino, Renata D’Incà, Salvatore Cucchiara, Maria Pastore, Mario D’Altilia, Daniela Scimeca, Giuseppe Biscaglia, Angelo Andriulli, Anna Latiano

**Affiliations:** 1 Division of Gastroenterology, “Casa Sollievo della Sofferenza” Hospital, IRCCS, San Giovanni Rotondo (FG), Italy; 2 Department of Surgical, Oncological, and Gastroenterological Sciences, University of Padua, Padua, Italy; 3 Department of Pediatrics, Pediatric Gastroenterology and Liver Unit, Sapienza University of Rome, Rome, Italy; 4 Division of Pediatrics, “Casa Sollievo della Sofferenza” Hospital, IRCCS, San Giovanni Rotondo (FG), Italy; Case Western Reserve University, UNITED STATES

## Abstract

**Background:**

Crohn’s disease (CD) is a pathologic condition with different clinical expressions that may reflect an interplay between genetics and environmental factors. Recently, it has been highlighted that three genetic markers, NOD2, MHC and MST1, were associated to distinct CD sites, supporting the concept that genetic variations may contribute to localize CD. Genetic markers, previously shown to be associated with inflammatory bowel disease (IBD), were tested in CD patients with the aim to better dissect the genetic relationship between ileal, ileocolonic and colonic CD and ascertain whether a different genetic background would support the three disease sites as independent entities.

**Methods:**

A panel of 29 SNPs of 19 IBD loci were analyzed by TaqMan SNP allelic discrimination method both evaluating their distinct contribute and analyzing all markers jointly.

**Results:**

Seven hundred and eight CD patients and 537 healthy controls were included in the study. Of the overall population of patients, 237 patients had an ileal involvement (L1), 171 a colonic localization (L2), and the 300 remaining an ileocolon location (L3). We confirmed the association for 23 of 29 variations (P < 0.05). Compared to healthy controls, 16 variations emerged as associated to an ileum disease, 7 with a colonic disease and 14 with an ileocolonic site (P < 0.05). Comparing ileum to colonic CD, 5 SNPs (17%) were differentially associated (P < 0.05). A genetic model score that aggregated the risks of 23 SNPs and their odds ratios (ORs), yielded an Area Under the Curve (AUC) of 0.70 for the overall CD patients. By analyzing each CD location, the AUC remained at the same level for the ileal and ileocolonic sites (0.73 and 0.72, respectively), but dropped to a 0,66 value in patients with colon localization.

**Conclusions:**

Our findings reaffirm the existence of at least three different subgroups of CD patients, with a genetic signature distinctive for the three main CD sites.

## Introduction

Crohn’s disease (CD) is a pathologic condition with different clinical expressions that may likely reflect a peculiar interplay between genetics and the environmental factors [1,2].

To date, more than 180 genes or loci have been associated with the susceptibility to CD [3–5]. Of these, 138 loci conferred the risk to both CD and ulcerative colitis (UC), whereas 42 were unique to CD, albeit each variant had a small individual effect. Major established determinants of the clinical course of CD are disease location, clinical behavior, age at disease onset, and extra intestinal manifestations [6–13]. While disease behavior and extraintestinal manifestation dramatically change over the course of CD, disease sites shows little or no variation [14] and might serve as a clue to define distinct entities of CD. Recently, Cleynen I. et al [15] highlighted three genetic markers (16q12/NOD2, 6p21/MHC and 3p21/MST1) associated to distinct CD sites, supporting the concept that peculiar genetic pathways may contribute to localize CD. From the genetic analysis, colonic CD had peculiar loci different from those expressed in either ileal CD and UC. Albeit the prospect to use genetic markers in refining the molecular classification of CD has been extensively evaluated, only the association between NOD2 variants and ileal CD has been validated unambiguously [16].

In the present work we tested a panel of 29 SNPs of 19 loci previously shown to be associated with disease sites of inflammatory bowel disease (IBD) to better dissect the genetic relationship between ileal, ileocolonic and colonic Crohn’s disease and ascertain whether a different genetic background would support the three disease sites as independent entities both evaluating their distinct contribute and analyzing all markers jointly.

## Subjects and Methods

### Patients

Seven hundreds and 8 CD cases were recruited at gastroenterologic and paediatric referral centres in the framework of the IBD genetic study, a multi-centre collaborative effort started in 1998 and co-ordinated by the Division of Gastroenterology of the IRCCS, ‘Casa Sollievo della Sofferenza’, Hospital, Italy. 403 DNA samples from CD patients were shared with the International IBD Genetic Consortium projects, and were also included in our previously works. 537 healthy blood donors without personal or familial history of inflammatory disorders were also included in the study. Patients’ feature and clinical data were stored in an anonymized database. The study and the experimental protocols received the approval from the ethic committee of the ‘Casa Sollievo della Sofferenza’ Hospital (N. 12701/08) and were performed in accordance with declaration of Helsinki approved guidelines. All participants had signed an informed consent form before study entry.

Diagnosis of CD was based on the Lennard Jones criteria [17]. According to Montreal’s classification [9] disease confined exclusively to the distal ileum, with or without cecum involvement, was labeled as L1, exclusively to the colon as L2, and as L3 in the event of an ilecolonic location. Disease located in the upper digestive tract was labeled as L4.

### Genotyping

DNA was extracted from peripheral blood using the Qiagen DNA Blood Kit procedure (Qiagen, Hilden, Germany). All patients and controls were genotyped for 29 Single Nucleotide Polymorphisms (SNPs) of 19 loci, using TaqMan SNP allelic discrimination method by means of an AB17900HT sequence detection system (Applied Biosystems Inc., Foster City, CA) at “Casa Sollievo della Sofferenza” Hospital, San Giovanni Rotondo, Italy.

The list of the investigated SNPs was obtained by screening of the available literature by two investigators (OP, AL). 26 CD-related SNPs encompassing 18 genomic loci from fine mapping or GWAs studies, were selected (S1 Table). Finally, we selected three variations among those identified by Cleynen et colleagues [15] encompassing the HLA locus: the SNPs rs6930777 and rs9268832, the SNP rs9267798 was typed instead of the rs4151651 being in Linkage Disequilibrium with the former (D’ = 0.82 and r^2^ = 0.62 in CEU population) (S1 Table).

### Statistical analyses

Univariate and multivariate stepwise logistic regressions were performed using SPSS software version 14.0 (SPSS, Chicago, IL, USA) and Haploview Software version 4.1 (http://www.broad.mit.edu/personal/mpg/haploview). The allelic frequencies for all investigated polymorphisms were tested for consistency with the Hardy-Weinberg equilibrium. Allelic and genotypic associations of SNPs were evaluated by Pearson's χ^2^ test (or Fisher’s test whenever appropriate). Compared to the control group, Odds ratio and 95% confidence intervals (CI) were estimated for each disease site (ileal, ileocolonic and colonic).

The stepwise logistic regression model was applied for all the variations significantly associated to the trait after correcting for multiple comparisons. This approach allowed to take into account a dose-response effect (heterozygote or homozygote), and the possible interactions between genes. P-values of less than 0.05 and 2.6e^-3^ after Bonferroni correction for multiple testing were considered significant.

In addition, to estimate the predictive value of multiple susceptibility loci on the disease status, we constructed a Genetic Risk Score (GRS) based on significant SNPs in the case-control study (P < 0.05) and their respectively odds ratios. We assigned to each subject a score based on the number of risk alleles carried for the SNPs associated with CD risk. We named “0” the common allele homozygote carriers, “1” the heterozygotes, and “2” the rare allele homozygotes. The number of risk alleles at each locus (2, 1, 0) was multiplied by their corresponding beta-coefficients of effect sizes [log(OR)] and then summed up in GRS that each individual carried. For the ORs we used “positive” scores achieved by the CD and healthy controls cohorts [18]. The receiving operating characteristic (ROC) curve was used to measure the area under the curve (AUC), sensitivity and specificity at various GRS cut-off.

## Results

### Case–control study

The allele frequencies of the SNPs analyzed were in accordance with the predicted Hardy-Weinberg equilibrium in all subgroups of CD (ileal CD, ileocolonic CD and colonic CD) (P > 0.05). Clinical and demographic characteristics of CD patients their initial presentation are shown in Table 1.

**Table 1:** Clinical and demographic characteristics of CD patients at initial presentation. Data are shown either for the entire cohort of patients and separately for disease sites.

	All		Disease sites					
	n = 708		*ileum (L1)*		*ileo-colon (L3)*		*colon (L2)*	
			n = 237		n = 300		n = 171	
	n	%	n	%	n	%	n	%
***GENDER***								
female	294	42	88	37	120	40	86	50
male	414	58	149	63	180	60	85	50
***Age at diagnosis (years)***								
mean±sd;	30±14		32±14		28±13		32±15	
***Age decades (years)***								
≤ 16	115	16	29	12	60	20	26	15
17-40	442	63	150	63	186	62	106	62
> 40	151	21	58	25	54	18	39	23
***Smoking***								
yes	218	31	76	32	95	32	47	27
no	366	53	114	48	156	52	96	56
ex	108	16	38	16	43	14	27	16
missing	16		9		6		1	
***Behavoiur***								
B1	537	76	164	69	224	75	149	87
B2	102	14	47	20	44	15	11	6
B3	69	10	26	11	32	10	11	6
***Familiar CD***								
yes	66	10	24	10	29	10	13	8
no	627	90	208	90	267	90	152	92
missing	15		5		4		6	
***Surgical presentation***								
yes	88	13	41	18	37	13	10	6
no	574	87	186	82	238	87	150	94
missing	46		10		25		11	
***Perianal disease***								
yes	15	2	4	2	8	3	3	2
no	683	98	229	98	290	97	164	98
missing	10		4		2		4	
***Colocation in upper digestive site***								
yes	47	7	17	7	25	8	5	3
no	661	93	220	93	275	92	166	97

### Association between polymorphisms and CD

The allele frequencies of the 29 SNPs analyzed were compared between CD patients and controls, and the results are reported in Table 2. We confirmed the association for 23 of the 26 selected variations associated to CD, while none of three SNPs located in the HLA region was significantly associated with CD. The most significant associations were observed for the variations rs2066844 [P = 1.0e^-5^, OR 2.1 (1.5–1.3)], rs2066845 [P = 9.0e^-8^, OR 4.0 (2.3–6.9)], and rs2066847 [P = 1.4e^-6^, OR 2.9 (1.8–4.6)] in the NOD2 gene, rs7517847 [P = 4.2e^-7^, OR 0.6 (0.5–0.8)] in IL23R gene, and rs1000113 [P = 4.3e^-6^, OR 1.7 (1.4–2.2)] in the IRGM gene.

**Table 2:** Allelic analysis in Crohn’s disease patients compared with healthy controls. * SNP associated after Bonferroni correction. Chr = Chromosome; SNP = single nucleotide polymorphism; RAF = Risk Allele Frequencies; OR = Odd Ratio; CI = confidence interval

			RAF				
Chr	SNP	KEY GENE	CASE,CONTROL		P values	OR	IC 95%
1p31.3	rs7517847	IL23R	0.72, 0.62	*	4.16E-07	0.6	0.5-0.8
1p31.3	rs11209026	IL23R	0.96, 0.93		4.95E-03	0.6	0.4-0.9
1p36.23	rs2797685	PER3	0.23, 0.19		7.30E-03	1.3	1.1-1.6
1q32.1	rs3024505	IL10	0.16, 0.14		0.13576	-	-
2p21	rs10495903	THADA	0.13, 0.10		2.39E-02	1.4	1.1-1.7
2p23.3	rs780093	GCKR	0.54, 0.53		0.53107	-	-
2q37.1	rs2241880	ATG16L1	0.59, 0.53		6.85E-03	1.3	1.1-1.5
3p21.31	rs9858542	BSN	0.38, 0.30		6.17E-03	1.3	1.1-1.6
3p21.31	rs3197999	MST1	0.34, 0.29		2.16E-02	1.2	1.2-1.5
5q31	rs2631367	SLC22A4	0.46, 0.41		3.89E-02	1.2	1.0-1.4
5q31	rs1050152	SLC22A5	0.52, 0.47		2.04E-02	1.2	1.0-1.4
5q31	rs11739135	IGR2198	0.44, 0.40		3.56E-02	1.2	1.0-1.4
5q31	rs1521868	IGR2196	0.46, 0.39	*	2.12E-03	1.3	1.1-1.5
5q33.1	rs1000113	IRGM	0.18, 0.11	*	4.30E-06	1.7	1.4-2.2
5q33.1	rs4958847	IRGM	0.22, 0.17	*	9.30E-04	1.4	1.2-1.8
6p21	rs9268832	MHC	0.77, 0.73		0.17819	-	-
6p21	rs6930777	MHC	0.93, 0.92		0.90981	-	-
6p21	rs9267798	MHC	0.07, 0.07		0.95427	-	-
6q25.3	rs212388	TAGAP	0.42, 0.41		0.52571	-	-
9q32	rs4263839	TNFSF15	0.76, 0.68	*	1.00E-05	0.7	0.5-0.8
10q21.2	rs10761659	ZNF365	0.55, 0.47	*	8.40E-04	1.3	1.1-1.6
10q22.3	rs150550	ZMIZ1	0.79, 0.75		2.58E-02	0.8	0.7-1.0
10q24.2	rs11190140	NKX2/3	0.57, 0.51		3.58E-03	0.8	0.7-0.9
16q12.1	rs2066844	NOD2	0.09, 0.04	*	1.00E-05	2.1	1.5-3.0
16q12.1	rs2066845	NOD2	0.06, 0.01	*	9.01E-08	3.8	2.3-6.9
16q12.1	rs2066847	NOD2	0.06, 0.02	*	1.37E-06	2.9	2.9-4.6
18p11.21	rs2542151	PTPN2	0.16, 0.12		8.41E-03	1.4	1.1-1.8
22q12.2	rs713875	MTMR3	0.54, 0.48		5.43E-03	1.3	1.1-1.5
22q13.1	rs2413583	MAP3K7IP1	0.84, 0.77	*	7.00E-05	0.7	0.5-0.8

Significant associations were achieved for the SNPs rs4263839 [P = 1.0e^-5^, OR 1.5 (1.3–1.8)] located in TNFSF15 gene and for the SNP rs2413583 [*P* = 7.0e^-5^, OR 1.5 (1.2–1.9)] near the MAP3K7IP1 gene. The SNPs located in loci encompassing the genes IL10, GCRK and TAGAP didn’t reach the nominal statistical significance.

### Ileum localization

When compared to healthy controls, 16 variations of the 29 analyzed SNPs (55%) emerged as associated to a disease localized to the ileum (P < 0.05) (Table 3). All these variants were referred to 12 loci. The NOD2 and MAP3K7IP1 genes, belonging to the innate immunity pathway, showed the strongest association. Carriers of the risk genotypes (aa + Aa) of the three NOD2 polymorphisms showed a highly statistical significance: rs2066844 [P = 1.4e^-6^, OR 2.8 (1.8–4.3)], rs2066845 [P = 1.2e^-5^, OR 3.8 (2.0–7.1)], and rs2066847 [P = 6.5e^-7^, OR 3.7 (2.1–6.4)]. At the univariate analysis, the presence of at least one of the NOD2 variants conferred a high risk for a disease confined to the ileum [P = 9.6e^-15^, OR 3.8 (2.7–5.3)]. Similarly, carriers of the variations rs1000113 and rs4958847 in the IRGM gene and of the rs713875 polymorphism of the MTMR3 gene, were at an increased risk for ileal CD: [P = 6.0e^-5^, OR 2.0 (1.5–2.9)], [P = 0.005, OR 1.6 (1.1–2.2)] and [P = 0.003, OR 1.8 (1.2–2.7)], respectively. All the remaining SNPs conferred the risk to an ileal localization. In particular, markers on the 3p21 locus named rs9858542 (BSN) and rs3197999 (MST1) manifested the highest association: [P = 0.003, OR 1.6 (1.1–2.4)], and [P = 0.02, OR 1.5 (1.1–2.1)], respectively. Some variants had a protective effect towards a CD localized to the ileum. This was the case for carriers of the minor genotype (aa) for rs2413583 on MAP3K7IP1 locus [*P* = 0.003, OR 0.2 (0.04–0.6)], and for those carrying the ‘aa’ genotype of rs7547847 located in the IL23R gene [P = 0.001, OR 0.4 (0.2–0.7)].

**Table 3:** Genotype analysis in ileal Crohn’s disease, colonic Crohn’s disease and ileocolonic Crohn’s disease patients. * SNP associated after Bonferroni corrections. SNP = single nucleotide polymorphism; OR = Odd Ratio; CI = confidence interval.

			ileum vs control	Colon vs control		ileocolon vs control		Ileum vs colon		ileocolon vs colon	ileocolon vs ileum
			P value	P value		P value		P value		P value	P value
SNP	KEY GENE		OR (95% CI)	OR (95% CI)		OR (95% CI)		OR (95% CI)		OR (95% CI)	OR (95% CI)
rs7517847	IL23R	*	1.3E-03	4.8E-03		4.8E-03					
			0.4 (0.2-0.7)	0.4 (0.2-0.8)		0.5 (0.3-0.8)					
rs11209026	IL23R					3.1E-03					
						0.5 (0.3-0.8)					
rs2797685	PER3		2.1E-02								
			1.5 (1.1-2.0)								
rs10495903	THADA		2.5E-02			3.8E-02					
			1.5 (1.1-2.2)			1.4 (1.0-2.1)					
rs2241880	ATG16L1		1.1E-02			1.3E-02					
			1.7 (1.1-2.6)			1.6 (1.1-2.3)					
rs9858542	BSN	*	2.6E-03	2.2E-02							
			1.7 (1.2-2.4)	1.6 (1.1-2.3)							
rs3197999	MST1		1.9E-02	7.5E-03							
			1.5 (1.1-2.1)	1.7 (1.1-2.4)							
rs2631367	SLC22A4		1.8E-02								
			1.6 (1.1-2.4)								
rs1000113	IRGM	*	6.0E-05	2.8E-02	*	2.3E-06					
			2.0 (1.4-2.9)	1.6 (1.1-2.3)		2.2 (1.6-3.0)					
rs4958847	IRGM		5.3E-03		*	9.8E-04					
			1.6 (1.1-2.2)			1.7 (1.2-2.3)					
rs4263839	TNFSF15				*	9.4E-04				3.9E-02	
						0.3 (0.2-0.7)				0.43 (0.19-0.98)	
rs10761659	ZNF365			5.5E-03	*	1.4E-03					4.7E-02
				1.9 (1.2-3.0)		1.8 (1.3-2.6)					1.53 (1.00-2.34)
rs150550	ZMIZ1		2.2E-02								
			2.9 (1.1-7.5)								
rs11190140	NKX2/3			1.1E-02		1.0E-02		2.6E-02			3.4E-02
				0.5 (0.3-0.9)		0.6 (0.4-0.9)		0.5 (0.3-0.9)			0.61 (0.39-0.97)
rs2066844	NOD2	*	1.4E-06		*	2.1E-03		2.4E-02			
			2.8 (1.8-4.3)			1.9 (1.3-3.0)		1.9 (1.1-3.2)			
rs2066845	NOD2	*	1.2E-05	2.7E-02	*	4.3E-09				2.2E-02	
			3.8 (2.0-7.1)	2.3 (1.1-4.9)		4.9 (2.8-8.9)				2.16 (1.10-4.22)	
rs2066847	NOD2	*	6.5E-07		*	3.7E-06	*	7.2E-05	*	2.2E-04	
			3.7 (2.1-6.4)			3.3 (1.9-5.6)		5.7 (2.2-15.0)		5.11 (1.98-13.21)	
At least one risk genotype	NOD2	*	9.6E-15		*	5.0E-11	*	6.2E-06	*	4,2E-04	
			3.8 (2.7-5.3)			2.9 (2.1-4.1)		2.8 (1.8-4.4)		2.19 (1.41-3.39)	
rs713875	MTMR3		2.7E-03			3.1E-02		4.4E-02			
			1.8 (1.2-2.7)			1.5 (1.0-2.1)		1.7 (1.0-2.8)			
rs2413583	MAP3K7IP1		3.4 E-03					2.1E-02			
			0.2 (0.04-0.6)					0.2 (0.04-0.9)			

Further, we applied a multiple stepwise logistic regression method for all the variations remaining significantly associated after using the Bonferroni correction for multiple comparisons (p<2.6e^-3^). For the NOD2, the presence of at least 1 variant was taken into account. All the variations resulted independently associated with ileal localization. In particular, the carriers of at least one of the NOD2 mutations [P = 6.9e^-10^, OR 3.8 (2.5–5.8)], as well as the carriers of rs9858542 (BSN) [P = 6.6e^-5^, OR 2.2 (1.5–3.3)], and rs10000113 (IRGM) [P = 4.7e^-4^, OR 2.1 (1.4–3.2)] mutations were at risk of developing an ileal disease, while those carried the “aa” genotype in rs7517847 (IL23R) resulted protected toward developing an ileal involvement [P = 0.01, OR 0.4 (0.2–0.8)] (data not shown). No evidence for statistical interactions between one gene to others were observed (*P* > 0.05).

### Colonic localization

When compared to healthy controls, seven (24%) SNPs of 7 loci resulted associated with a colonic disease (Table 3). The most relevant association was for variations in genes IL23R, MST1 and for those on the locus 10q21. In particular, the ‘aa’ genotype of the rs7517847 (IL23R) conferred protection [*P* = 0.005, OR 0.40 (0.2–0.8)], while carriers of the ‘AA’ or ‘Aa’ genotypes of the variations rs10076169 (ZNF365) and rs3197999 (MST1) were at higher risk: [P = 0.005, OR 1.9 (1.2–3.0)] and [P = 0.007, OR 1.9 (1.1–2.4)], respectively. After applying the Bonferroni correction, all prior associations were nullified.

### Ileo-colon localization

For this disease site, 14 SNPs (48%), all belonging to 9 loci, emerged as significantly associated (Table 3). The polymorphisms of the NOD2 showed the highest association: rs2066844 [P = 0.002, OR 1.9 (1.3–3.0)], rs2066845 [P = 4.3e^-9^, OR 5.0 (2.8–8.9)] and rs2066847 [P = 3.7e^-6^, OR 3.3 (1.9–5.6)]. Carriers of at least one of the previous mutations were at risk for the ileocolonic disease [P = 5.0e^-11^, OR 2.9 (2.1–4.1)]. The two variations in the IRGM gene were both associated to ileocolonic localization namely the rs1000113 [P = 2.3e^-6^, OR 2.2 (1.6–3.1)], and the rs4958847 [P = 0.001, OR 1.7 (1.2–2.3)]. The SNP rs10761659 of the ZNF365 gene was also associated with this CD site [P = 0.001, OR 1.6 (1.1–2.3)].

A protective effect toward a CD located simultaneously to the colon and the ileum was afforded by two SNPs of the IL23R gene, namely rs11209026 [P = 0.003, OR 0.5 (0.3–0.8)] and rs7517847 [P = 0.005, OR 0.5 (0.3–0.8)]; the SNP rs4263839 of the TNFSF15 gene was also associated with this disease site [P = 0.001, OR 0.3 (0.2–0.7)]. At multiple stepwise logistic regression analysis, all the variations, at exception of the rs4958847 in the IRGM gene, were independent predictors of an ileocolonic localization. In particular, carriers of at least one NOD2 mutations [P = 2.1e^-7^, OR 2.9 (1.9–4.3)], were at risk for the colonic disease, as well as the carriers of mutations in rs10761659 (ZNF365) [P = 6.6e^-4^, OR 2.1 (1.4–3.1)] and those in rs10000113 (IRGM) [P = 2.8e^-4^, OR 2.0 (1.4–3.0)]. Carriers of the “aa” genotype in rs4263839 (TNFSF15) resulted protected to develop a ileocolonic disease [P = 0.04, OR 0.4 (0.2–0.9)] (data not shown). No evidence for statistical interaction between these genes was observed (*P*>0.05).

### Ileum *vs* colonic localization

By comparing the ileal to the colonic CD, 5 out of 29 SNPs (17%), belonging to 4 loci, were differentially expressed (P < 0.05) (Table 3). In particular, the significant association was shown in patients carrying the NOD2 risk genotypes ‘aa’ + ‘Aa’ at either the rs2066844 [*P* = 0.02, OR 1.9 (1.1–3.2)] or the rs2066847 [*P* = 7.2e^-5^, OR 5.8 (2.2–15.0)] and in those with at least one of the 3 NOD2 markers [*P* = 6.2e^-6^, OR 2.8 (1.8–4.4)]. In contrast, the ‘aa’ genotype for rs2413583 (MAP3K7IP1) was proctective for the ileal localization [*P* = 0.02, OR 0.2 (0.04–0.9)].

### Ileocolon *vs* colonic localization

By comparing the two disease sites, 3 SNPs (10%) emerged as significantly associated (Table 3), with SNP rs2066847of the NOD2 gene revealing the highest association [P = 2.2e^-4^, OR 5.1 (2.0–13.2)]. Carriers of at least one of the 3 NOD2 mutations were at risk for the ileocolonic disease [P = 4.2e^-6^, OR 2.2 (1.4–3.4)]. In addition, SNP rs4263839 of the TNFSF15 confers protection for an ileocolon localizzation [P = 0.04, OR 0.43 (0.2–1.0)].

### Ileocolon *vs* ileal localization

Only 2 (7%) variations were statistically different between ileocolonic and ileal localizations: SNP rs10761659 of the ZNF365 gene conferred an increased risk [P = 0.047, OR 1.5 (1.0–2.3)], and the variation rs11190140 of the NKX2/3 [P = 0.04, OR 0.6 (0.1–1.0)] showing a protective effect for the ileocolonic localization (Table 3). Either in NOD2 positive and negative patients, the frequency of smokers and non-smokers was weakly significant (P = 0.067) (data not shown).

### Cumulative genetic risk score

**Fig 1:**
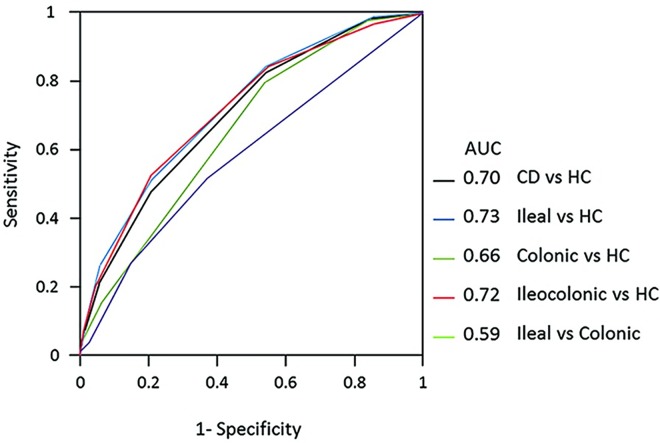
Receiver Operating Characteristic curve (ROC) for allele count and weighted genetic risk score (GRS) based on 23 SNPs used to measure the area under the curve (AUC) in ileal Crohn’s disease, colonic Crohn’s disease and ileocolonic Crohn’s disease patients compared to healthy controls, and in ileal Crohn’s disease compared to colonic Crohn’s disease patients.

We aggregated the information from the 23 genetic variants associated to CD and combined the contribution of each nucleotide polymorphism into a genetic risk score (GRS) (Fig 1). In the whole CD cohort, the Area Under the Curve (AUC) was 0.70, using a cut off value of 5.5 alleles (P = 8.6^e-20^; sensitivity = 0.83; specificity = 0.46). By analyzing each Crohn’s disease location versus healthy controls, the AUC increased in patients with ileal (AUC = 0.73; *P* = 5.4e^-13^; sensitivity = 0.85; specificity = 0.46) and ileocolonic localization (AUC = 0.72; *P* = 3.2e^-14^; sensitivity = 0.83; specificity = 0.46); in patients with pure colon localization an AUC of 0.66 was found (P = 1.4e-6; sensitivity = 0.80; specificity = 0.46). Next we considered the differentiating ability between an ileal or a colonic disease after combining the 23 genetic markers significantly associated to CD in the present investigation. The resulting GRS performed poorly in differentiating colonic versus ileal disease: AUC = 0.58; P = 0.03. Improved results became evident when we considered in the analysis only the NOD2 (rs2066844 and rs2066847), NKX2/3, MAP3K7IP1 and MTMR3 SNPs: AUC = 0.60; P = 2.7e^-3^; sensitivity = 0.52; specificity = 0.65 (data not shown).

## Discussion

Genome-wide association studies, Immunochip and meta-analyses identified in patients with CD different susceptibility loci and possible candidate genes, that might affect the predisposition to the disease. Most involved genes were related to the innate and adaptive immunity, bacterial recognition, and autophagy-related mechanism. Cumulative evidences support the concept that deficiencies of innate immune cell functions, principally due to the three major mutations in the NOD2 gene, represent a crucial key in CD and may help distinguish it from UC [15, 19].On the other side, serological and fecal biomarkers, although have the potential to become cornerstones of predictive models for monitoring the course of IBD, have been inconsistent in distinguishing either patients with CD or UC or in determining the different intestinal location of CD [20].

Very recently, a large genotype-phenotype study, carried out by the International IBD Genetic Consortium, highlighted that three loci namely 16q12/NOD2, 6p21/MHC and 3p21/MST1 were of some guide in conditioning the site of CD within the gastrointestinal tract, with the 16q12/NOD2 locus as a major determinant of ileal disease, and the 6p21/MHC locus mainly associated with colonic disease [15].

The aim of the present work was to assess the potential advantage to use a panel of genetic markers to predict the clinical location in CD patients, and to use this information to stratify patients for a more effective targeted medical treatment.

In a case control study, we confirmed the association for 23 of the 26 variations associated to CD and IBD, while none of the HLA markers suggested in the Cleynen [15], resulted significantly associated. As expected, the most significant associations were observed for the variants in NOD2, IL23R, IRGM, TNFSF15 and MAP3K7IP1 genes.

Considering the three NOD2 major variants, the strongest association was achieved in carriers of at least one mutation. These figures remained still significant when we sorted the entire cohort of CD patients by ileal and Ileocolonic disease sites as compared to healthy controls. Carriers of at least one risk genotype of the L1007fsX (rs2066847) variant were statistically associated to ileal localization versus the colonic ones. This variant is also able to discriminate between ileocolonic and colonic sites increasing the risk in patients with ileocolonic CD. In accordance with Cleynen paper [15], NOD2 gene drives the association with both ileal and ileocolonic disease location, and L1007fsX variant remained the best marker for both sites. The smoking habits resulted only weakly associated in the group of NOD2 negative patients with ileal involvement, although smoking is one of the most replicated risk factors in CD pathogenesis [2].

A significant association was also found for the IRGM gene, that plays a role in the innate immune response by regulating autophagy in response to intracellular pathogens. Two polymorphisms of the IRGM gene were associated with CD risk in the study by the Wellcome Trust Case–Control Consortium GWA [21]. In addition, in a previous study we found these polymorphisms significantly associated with an aggressive behavior of CD [22]. In the present investigation, we confirmed the data for the two variations of the IRGM gene. In detail, for the rs1000113 variant of this gene we found a strong association with the ileal and ileocolon localization. Moreover, for the rs4958847 variant the association power was even higher in patients with ileocolonic localization. We have to acknowledge that after applying the Bonferroni correction, the significance of these associations waned off.

Of interest, the genes analyzed in this study, namely the TNFSF15 and MAP3K7IP1, proved to have a protective power for the development of CD. The original observation that the TNFSF15 gene was strongly associated with CD was provided by studying a Japanese cohort of patients and our Italian cohort [23, 24]. The gene encodes for a protein named TL1A which exerts a defensive role in the gut against pathogens [25, 26]. We found the rs4263839 variation of this gene to be associated to CD cohort: carriers of the ‘aa’ genotype appeared to be protected against the development of the disease at colonic and ileocolonic site, but not at the ileal site. On the contrary, the rs2413583 SNP of the MAP3K7IP1 gene was protective for an ileal CD. This variation was associated in CD by Franke et coll. [27]. Our investigation is the first independent confirmation of the protective power of the marker rs2413583 to development an ileal CD.

In the present investigation we typed 2 SNPs on the locus 3p21 encompassing the genes BSN and MST1. This locus resulted one of the three loci associated in the genotype-phenotype study by the International Consortium [15] and influenced the occurrence of extraintestinal manifestations in CD patients in our previously work [28]. Both markers resulted associated with CD, although only rs9858542 persisted associated with ileal localization following the Bonferroni correction; the significance was still maintained at the multiple stepwise logistic regression analysis. On the contrary, any association with SNPs of the HLA region was detected in our CD population. These data are in contrast with Cleynen study [15], where HLA markers prevailed in colonic CD. The numerically small sample size of the present study may have obscured the HLA impact on colonic CD.

The value of including all information provided by the single associated variants to CD in a genotype risk score was an essential component of this study even if the emerging results appear of marginal clinical benefit. Indeed, our genotype risk score, based on 23 single variations, proved to have a discriminant capacity for the diagnosing CD at a statistically level, even if the AUC value was only 0.70 (*P* = 8.6e^-20^). The AUC value increased minimally when restricting the analysis to patients with pure ileum (AUC = 0.73; *P* = 5.4e^-13^) or ileocolon (AUC = 0.72; *P* = 3.2e^-14^) involvement as compared to the control group, but the AUC value decreased in those with colonic involvement (AUC = 0.66; *P* = 1.4e^-6^), well in keeping with similar results from previous investigations [29–33].

To date, there are not available genetic markers that can be used to accurately predict the risk of developing Crohn's disease. Here we emphasize that the markers, so far identified as associated with CD, are actually more specifically linked to an ileocolonic and an ileal location of CD disease, whereas the pure colon CD remains orphan of a substantial genetic support.

In conclusion, although with the paucity of panel of loci analyzed, our findings reaffirm the existence of at least three different subgroups of CD patients, with a genetic signature distinctive to the three main CD sites. These results might have therapeutic implications in future work in the event a specific therapeutic target will be proved more effective in specific sites of the CD in respect to the other one(s).

## Supporting Information

S1 Table List of analyzed variations. (DOCX)
