# Supplementary material for: Crohn’s Disease Localization Displays Different Predisposing Genetic Variants
Source: PLoS One. 2017 Jan 4;12(1):e0168821. doi: 10.1371/journal.pone.0168821 (PMC5215692; doi:10.1371/journal.pone.0168821)
Supplement: S1 Table — (DOCX) [file pone.0168821.s001.docx]

| Chr | SNP | KEY GENE | Reference |
| --- | --- | --- | --- |
| 1p31.3 | rs7517847 | IL23R | 1 |
| 1p31.3 | rs11209026 | IL23R | 1 |
| 1p36.23 | rs2797685 | PER3 | 2 |
| 1q32.1 | rs3024505 | IL10 | 3 |
| 2p21 | rs10495903 | THADA | 4 |
| 2p23.3 | rs780093 | GCKR | 4 |
| 2q37.1 | rs2241880 | ATG16L1 | 5 |
| 3p21.31 | rs9858542 | BSN | 4 |
| 3p21.31 | rs3197999 | MST1 | 4 |
| 5q31 | rs2631367 | SLC22A4 | 6 |
| 5q31 | rs1050152 | SLC22A5 | 6 |
| 5q31 | rs11739135 | IGR2198 | 7 |
| 5q31 | rs1521868 | IGR2196 | 7 |
| 5q33.1 | rs1000113 | IRGM | 8 |
| 5q33.1 | rs4958847 | IRGM | 8 |
| 6p21 | rs9268832 | MHC | 9 |
| 6p21 | rs6930777 | MHC | 9 |
| 6p21 | rs9267798 | MHC | 9 |
| 6q25.3 | rs212388 | TAGAP | 4 |
| 9q32 | rs4263839 | TNFSF15 | 10 |
| 10q21.2 | rs10761659 | ZNF365 | 4 |
| 10q22.3 | rs150550 | ZMIZ1 | 4 |
| 10q24.2 | rs11190140 | NKX2/3 | 8 |
| 16q12.1 | rs2066844 | NOD2 | 11 |
| 16q12.1 | rs2066845 | NOD2 | 11 |
| 16q12.1 | rs2066847 | NOD2 | 11 |
| 18p11.21 | rs2542151 | PTPN2 | 4 |
| 22q12.2 | rs713875 | MTMR3 | 4 |
| 22q13.1 | rs2413583 | MAP3K7IP1 | 2 |

**S1 Table:** List of analyzed variations.

Chr = Chromosome; SNP = single nucleotide polymorphism

**References**

1) Duerr RH, Taylor KD, Brant SR, Rioux JD, Silverberg MS, Daly MJ, et al. A genome-wide association study identifies IL23R as an inflammatory bowel disease gene. Science. 2006;314:1461-3.

2) Franke A, McGovern DP, Barrett JC, Wang K, Radford-Smith GL, Ahmad T et al. Genome-wide meta-analysis increases to 71 the number of confirmed Crohn's disease susceptibility loci. Nat Genet. 2010;42:1118-25.

3) Franke A, Balschun T, Karlsen TH, Sventoraityte J, Nikolaus S, Mayr G, et al. Sequence variants in IL10, ARPC2 and multiple other loci contribute to ulcerative colitis susceptibility. Nat Genet. 2008;40:1319-23.

4) Jostins L, Ripke S, Weersma RK, Duerr RH, McGovern DP, Hui KY,et al. Host-microbe interactions have shaped the genetic architecture of inflammatory bowel disease. Nature. 2012;491:119-24.

5) Hampe J, Franke A, Rosenstiel P, Till A, Teuber M, Huse K, et al. A genome-wide association scan of nonsynonymous SNPs identifies a susceptibility variant for Crohn disease in ATG16L1. Nat Genet. 2007;39:207–11.

6) Peltekova VD, Wintle RF, Rubin LA, Amos CI, Huang Q, Gu X, et al. Functional variants of OCTN cation transporter genes are associated with Crohn disease. Nat Genet. 2004;36:471-5.

7) Rioux JD, Daly MJ, Silverberg MS, Lindblad K, Steinhart H, Cohen Z, et al. Genetic variation in the 5q31 cytokine gene cluster confers susceptibility to Crohn disease. Nat Genet. 2001;29:223-8.

8) Wellcome Trust Case Control Consortium. Genome-wide association study of 14,000 cases of seven common diseases and 3,000 shared controls Nature. 2007;447:661-78.

9) Cleynen I, Boucher G, Jostins L, Schumm LP, Zeissig S, Ahmad T, et al. Inherited determinants of Crohn's disease and ulcerative colitis phenotypes: a genetic association study. Lancet. 2016;387:156-67.

10) Yamazaki K, McGovern D, Ragoussis J, Paolucci M, Butler H, Jewell D, et al. Single nucleotide polymorphisms in TNFSF15 confer susceptibility to Crohn’s disease. Hum Mol Genet 2005;14:3499–3506.

11) Hugot JP, Chamaillard M, Zouali H, Lesage S, Cézard JP, Belaiche J et al. Association of NOD2 leucine-rich repeat variants with susceptibility to Crohn's disease. Nature. 2001;411:599-603.
